# Public Attitudes towards Prevention of Obesity

**DOI:** 10.1371/journal.pone.0039325

**Published:** 2012-06-19

**Authors:** Claudia Sikorski, Melanie Luppa, Georg Schomerus, Perla Werner, Hans-Helmut König, Steffi G. Riedel-Heller

**Affiliations:** 1 Integrated Research and Treatment Center AdiposityDiseases, University of Leipzig, Leipzig, Germany; 2 Institute of Social Medicine, Occupational Health and Public Health, University of Leipzig, Leipzig, Germany; 3 Department of Psychiatry and Psychotherapy, University of Greifswald, Stralsund, Germany; 4 Faculty of Social Welfare and Health Sciences, University of Haifa, Haifa, Israel; 5 Department of Medical Sociology and Health Economics, Hamburg-Eppendorf University Medical Center, Hamburg, Germany; The University of Texas M. D. Anderson Cancer Center, United States of America

## Abstract

**Objective:**

To investigate obesity prevention support in the German general public and to assess determinants of general prevention support as well as support of specific prevention measures.

**Methods:**

This study was a cross-sectional analysis of a telephone based representative German study (3,003 subjects (52.8% women, mean age 51.9, s.d. = 18.0, range 18–97 years). Likert scale-based questions on general prevention support and support of specific measures were used. Furthermore willingness to take part in preventive programs and willingness to pay were assessed. Stigmatizing attitudes were assessed with the Fat Phobia Scale (FPS). Causation of obesity was differentiated in three dimensions (internal, e.g. lack of exercise; external, e.g. social surroundings; and genetic factors).

**Results:**

Obesity prevention was perceived as possible (98.2%), however, almost exclusively lifestyle changes were named. Participants with higher stigmatizing attitudes were less likely to believe obesity prevention is possible. The majority of participants would take part in preventive programs (59.6%) and pay at least partially themselves (86.9%). Factor analysis revealed three dimensions of preventive measures: promoting healthy eating, restrictive and financial, governmental prevention efforts. In regard to these, promoting healthy eating was the most supported measure. Higher age, female gender and external causation were associated with higher support for all three dimensions of preventive measures. Only for governmental regulation, higher age was associated with lower support.

**Conclusion:**

Obesity prevention support in Germany is high. Structural prevention efforts are supported by the majority of the general public in Germany. The vast majority proclaims willingness to pay themselves for programs of weight gain prevention. This could be an indication of higher perceived self-responsibility in the German system but also for risen “fear of fat” in the population due to media coverage. For Germany, the government and communities ought to be encouraged by these results to start the implementation of structural obesity prevention.

## Introduction

Obesity has become a major health problem in western countries and has also started to increase in developing countries. The International Obesity Taskforce estimates about 600 million people to be obese worldwide [1].

Health systems are faced with an enormous economic burden [2]; [3]. Already today, 7.5% of the entire disease burden measured in disability-adjusted life years (DALYs) in high income countries is caused by overweight and obesity [4]. For Germany, direct costs (health care provided for affected patients) cumulate up to 4.854 billion Euros which corresponds to 2.1% of all health expenditures in 2002. Indirect costs, incurred by productivity loss due to illness related work loss days and missed career opportunities, sum up to 5.019 billion Euros per year [2].

Having previously played only a minor role in building health care strategies in many countries, due to the excessive rise in prevalence rates and health care costs, obesity awareness and prevention are now becoming part of public health initiatives [5]; [6]. In the past, this development has led to higher media attention and undirected programs [7]; [8]. In light of financial restrictions within health care systems, however, a variety of laws, regulatory measures and public health efforts need to be applied [9] and funding for preventive strategies needs to be boosted [10].

Obesity prevention covers the range from primary prevention (health promotion prior to weight gain) to secondary prevention (preventing further weight gain in obese individuals). Action fields can either be aimed at individual behavior (e.g. exercising, dieting) or structural changes (e.g. laws etc.).

In publically funded health care and social security systems, such as the German one, justification of expenses for prevention efforts is eminent [11]. Especially in such settings, measures that are supported by the general public are needed. On a broader level, it is not only necessary to determine prevention support rates in the general public, but a thorough understanding of factors determining attitudes is also needed.

For example, the concept of full individual responsibility for obesity might result in lower support for prevention efforts [12]. Up to this date, a variety of individual-based measures has been proposed and evaluated with at most modest results in effectiveness [13]. Within societies that primarily attribute obesity to individual lifestyle behavior and choices [14] these interventions have dominated the field. However, a change of incentive structure by lowering costs for healthy behavior and raising costs of unhealthy behavior (“libertarian paternalism”) has been proposed [15].

This study therefore aims at enlightening obesity prevention support in the German public by also covering structural interventions. A representative sample was analyzed in order to answer the following questions: (1) Does the lay public consider prevention of obesity possible? (2) Would people take part in prevention programs, would they pay for it (and how much)? (3) What structural interventions are supported? (4) What variables on socio-demographic and condition-related levels are associated with attitudes towards prevention support?

## Methods

### Sample

In February 2011, a computer-assisted telephone interview (CATI) was conducted in a population-based sample of German residents by USUMA, a leading market, opinion and social research institute in Germany. Participants were selected using random digital dialing and Kish-Selection-Grid when choosing the person in the household [16]. In total 5 897 individuals were contacted from which 32.6% (n = 1,998) refused to participate. Another 16.5% could not be reached, reflecting a response rate of 50.9%. Respondents were informed verbally of the focus of the study and following publications in journals. The study was approved by the Ethics committee of the University of Leipzig (Ethik-Kommission an der Medizinischen Fakultät der Universität Leipzig). USUMA documented the consent and refusal of each participant within the CATI.

**Figure 1:**
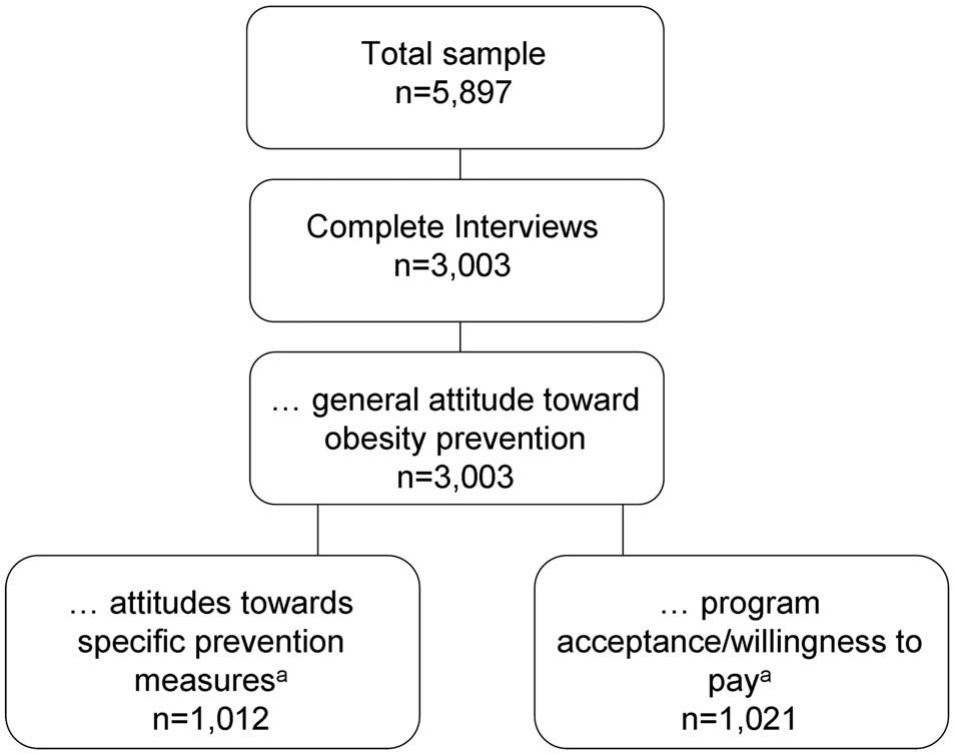
Number of participants included in analysis for the three different areas of prevention support. ^ a^randomly selected respondents.

The total sample comprises 3 003 persons. Due to time restrictions, parts of the interview were only assessed in smaller samples. Figure 1 shows sample sizes for each block of questions. Random selection for each block ensures representativeness of the German population. Table 1 displays socio-demographic characteristics of the samples compared to the German general population. Our sample contained slightly older and better educated citizens.

**Table 1:** Characteristics of the samples.

	Total Sample(n = 3 003)	Sub-sample I(n = 1 012)	Sub-sample II(n = 1 021)	German Population12/2009^1^
Women	52.8	52.3	53.4	51.0
Age group				
<20	4.9	4.8	4.2	18.8
21–40	22.4	24.1	22.1	24.3
41–60	37.2	36.8	38.7	31.0
60–80	31.5	29.6	30.5	20.8
>81	4.0	4.7	4.5	5.1
Education				
Student	1.2	1.2	0.6	3.5
8/9 yrs of schooling	23.7	22.1	23.2	37.0
10 yrs of schooling	32.2	31.3	34.5	28.8
12/13 yrs of schooling	42.4	44.9	41.0	25.8
No education	0.3	0.2	0.6	4.1

1 Federal Statistics Office (December 2009).

### Instruments

The study team conducted preliminary focus groups in order to develop items for the fully structured interview. Three focus groups with health care professionals, participants of the lay public and affected overweight individuals were held [17]. The following measures were assessed:

1. General attitude towards obesity prevention and condition-related characteristics
  We firstly asked respondents, what would be possible measures to prevent obesity. Answers were recorded and later transcribed. Furthermore, we inquired whether participants saw obesity as a problem that has to be solved individually or on a societal level (Likert scale 1–5). In order to assess personal experience with obesity, we recorded height and weight of the participants as well as whether they had an overweight partner.
2. Attitudes regarding possible prevention measures
  Based on qualitative analysis of the focus groups, we compiled a list of 14 preventive measures that were then presented to the interviewees. Within the focus groups no main themes were introduced. The interviewers asked them to rate each action on a Likert five-point rating scale from 1 (“oppose completely”) to 5 (“support completely”).
3. Program acceptance and willingness to pay
  This section included a question on willingness to take part in an obesity prevention program. Since we were able to determine body mass index (BMI) of participants, wording of the questions was altered when the participant was already overweight. The question then was: “Would you take part in a program to prevent further weight gain?” (yes/no). We then asked whether potential participants of these programs should either pay themselves, pay partly themselves or completely themselves for the participation. When participants agreed that the individuals ought to pay at least partly for the programs, we asked how much they would spend on such programs per year themselves. Five options ranging from “at most 20 Euro” to “more than 100 Euro” were offered.
4. Other relevant measures
  The interview also included questions on the stigma of obese fellow citizens. As done in previous research experimental manipulation was conducted using a vignette methodology [18]; [19]. Six different vignettes (male/female * child/adult/senior citizen) were introduced. Stigmatizing attitudes were assessed using a semantic differential in form of a German version of the Fat Phobia Scale (FPS) [20]. A mean FPS score ranging from 1 to 5 was calculated, with higher scores indicating higher negative attribution. Likewise, interviewees were asked to rate the importance of possible causes for the vignettes’ obesity. Fourteen items were presented and were rated from 1 (“not important at all”) to 5 (“highly important”). A factor analysis was conducted, yielding a three-factor solution (Eigenvalue >1). The factors can be labeled “individual causes” (such as quantity of food, willpower, lack of activity behavior), “external causes” (beyond the individuals’ control such as social environment, upbringing) and “genetic influences” (genetics and metabolism). A mean score for each factor was calculated.

### Data Analysis

After descriptive analyses, we investigated the influence of socio-demographic and condition-related characteristics on the named dimensions (general prevention attitudes, part-taking in programs and willingness to pay) with logistic regression. All outcome variables were dichotomized. Age, Gender, residence (former Eastern vs. Western part of Germany), high school education (12 years vs. less than 12 years) and BMI (continuous) served as independent variables. Additionally, views on the three factors of causal beliefs, the mean FPS score, the presence of an overweight partner and attitudes regarding the responsibility of obesity management (societal vs. individual) were introduced.

All specific prevention measures were entered into a principal component analysis in order to determine and confirm a potential underlying classification structure. The Kaiser-Meier-Olkin (KMO) measure of sampling adequacy was calculated for each of the 14 items. The KMO provides an indication of whether all variables are apt for inclusion in the factor analysis. If the KMO was below 0.8, items were omitted [21]. Factors with an Eigenvalue greater than 1 were retained and varimax rotated factor loadings of the items were calculated. Mean factor scores were determined and were then used as dependent variables in linear regression models. The same independent variables as in the logistic models were introduced. All models are complete models with simultaneous introduction of independent variables. In all analyses “no response” codes were treated as missing values. The sample on questions regarding willingness to pay includes participants with different vignettes, therefore making it necessary to control for vignette influences. All analyses were performed using STATA 11.2 [22].

## Results

The view on obesity prevention in the general public in Germany was mainly optimistic and positive. Of the 3 003 respondents, only 53 (1.76%) stated that there was no way to prevent obesity. The vast majority named a variety of measures that they believed to be an effective prevention measure. Most often named were individual and behavioral preventive strategies such as eating healthier (72%) and exercising more often (77%). Components that would be regarded as “healthier eating” are rarely named, e.g. a reduction of fast-food consumption was named by 1.4% of all respondents. Government based and structural prevention programs were rarely named; 17.8% plead for school and kindergarten based informational campaigns and only 1.3% thought to include the food industry in regards of labeling food. Out of 1 021 participants that were asked, 609 (59.6%) stated that they would be willing to partake in a prevention program. Numbers did not differ for overweight and normal-weight individuals (χ^2^ = 2.2438, p = 0.134). A majority would pay for those programs at least partly themselves (21% completely, 65.9% partially). Those, that stated they would cover expenses at least partially themselves and signaled willingness to participate in programs were than asked to estimate how much they would spend on a yearly basis. Only 4.4% would pay less than 20 € per year while more than two thirds of the population stated to be willing to pay between 50 and more than 100 € (69.0%).

Table 2 displays the results of logistic regression analyses. Socio-demographic and condition-related variables served as independent variables, investigating their association with positive attitudes towards obesity prevention. A general opinion that obesity prevention is not possible was associated with a higher score on the FPS, indicating a more negative view of obese fellow citizens. Willingness to take part in preventive programs was correlated with lower age and higher BMI. Also, attributing obesity to genetic factors was associated with a higher readiness to partake in programs. Regarding the openness to pay for the expenses of such programs, higher age was associated with a higher willingness to pay.

**Table 2:** Regression models on attitudes towards obesity prevention.

	Prevention is possible(yes/no) (n = 2 849)	Taking part in preventiveprograms (yes/no) (n = 972)	Paying for preventive programs (yes/no) (n = 961)
	OR (95% CI)	OR	OR
Age (years)	1.00 (0.98–1.01)	0.99** (0.98–1.00)	1.02** (1.01–1.03)
Female	0.82 (0.45–1.51)	1.33 (1.02–1.74)	1.07 (0.73–1.58)
Living in Eastern part of Germany	1.62 (0.83–3.16)	1.07 (0.76–1.46)	0.89 (0.55–1.40)
High school education (12 yrs vs. less)	*Dropped* a	*Dropped* a	1.11 (0.88–1.41)
External causation (mean agreement score)	0.76 (0.46–1.25)	1.16 (0.94–1.43)	0.79 (0.58–1.08)
Internal causation (mean agreement score)	1.21 (0.72–2.04)	0.99 (0.79–1.25)	1.24 (0.89–1.72)
Genetic causation (mean agreement score)	0.85 (0.59–1.22)	1.25** (1.07–1.47)	0.90 (0.71–1.13)
BMI (continuous)	1.05 (1.00–1.11)	1.04** (1.01–1.08)	1.00 (0.95–1.04)
Overweight partner (yes/no)	1.43 (0.55–3.71)	1.20 (0.72–2.01)	0.77 (0.39–1.54)
Problem solution^b^	1.06 (0.80–1.42)	1.08 (0.95–1.22)	0.98 (0.94–1.03)
Stigmatizing attitudes (FPS, mean score)	0.39** (0.20–0.77)	1.09 (0.81–1.47)	0.83 (0.54–1.27)
Pseudo R^2^ (%)	5.1	2.9	2.7

All variables simultaneously introduced, full models. Adjusted for vignette influences, *p<0.05, **p<0.01, ***p<0.001.

a Education was dropped due to multicollinearity;

b “Obesity is a problem that has to be solved individually ( = 1) or on a societal level ( = 5)”;

BMI – Body Mass Index, CI – confidence interval; FPS – Fat Phobia Scale; OR – Odds Ratio.

In table 3, all strategies that were presented to the interviewees are displayed. Support was defined as the two categories closest to the anchor point “strongly support”. Items in the spectrum of school-based prevention received highest approval rates. Participants supported tax benefits the least.

**Table 3:** Approval of prevention strategies for obesity (n = 1 012).

Strategy	Rated as helpful	
	n	(%)
Supplying students with healthy food/fruits	959	95.1
School curriculum on healthy eating and information	933	92.8
Establishing and optimizing nutrition labelling of foods	875	86.7
Educating parents on healthy eating	864	85.7
Campaigns for healthy eating	834	82.8
Banning of misleading advertisements	812	81.0
Restricting advertisements for unhealthy food on children’s TV channels	812	80.8
Broadcasting specific advertisements on healthy eating	780	77.5
Financial support/subvention of gym classes	772	76.7
Banning unhealthy food (fast food) and soft drinks from schools	758	75.2
Government based offers for active lifestyles	703	70.3
Health care insurance bonus for active/health beneficial activites	701	70.2
Restricting advertisements for unhealthy food	556	55.3
Tax benefits for expenses spent on sport and gym activities	508	51.0

The underlying factor structure was examined and confirmed by a factor analysis. Calculating the Kaiser-Meyer-Okin measure of sampling adequacy left 13 items to be introduced into the factor analysis. In a next step, two items had to be removed due to low factor loading below 0.40. Three factors with an Eigenvalue greater than 1 were extracted. Table 4 depicts varimax rotated factor loadings of the remaining 11 items. Factor 1 was termed “healthy eating promotion”, including items that focused on alteration of eating habits (4 items). Factor 2 was named “restriction measures” as it included items on banning (4 items). The last factor was named “financial, governmental regulation”, listing measures that directly included the government and financial aspects (3 items). The item “campaigns on health eating” had moderate loadings on factor one and three but fit into factor one content wise. The three factors accounted for 55.1% of the variance. The factor scores were then regressed on socio-demographic and illness related variables. Results are displayed in table 5. Generally, women and older participants were more in favor of each prevention strategy. Variables associated with higher approval of healthy eating related prevention were an attribution of obesity to external and internal factors. Support for banning and restricting e.g. advertisement was higher in those living in the former Eastern part of Germany. Additionally, a higher external causation belief was associated with higher approval rates. Factor 3 (monetary and governmental regulation) was associated with external and internal causation beliefs as well as residence in the former Eastern part of Germany. Higher stigmatizing attitudes led to higher support. Explained variance for the three models was 10.0%, 9.0% and 8.5% respectively.

**Table 4:** Underlying structure in prevention measures.

Variable	Factor 1(healthy eating promotion)	Factor 2(restriction)	Factor 3(financial, governmental regulation)
School curriculum on healthy eating and information	**0.7901**	0.1782	0.0508
Supplying students with healthy food/fruits	**0.6257**	0.2007	0.2225
Educating parents on healthy eating	**0.7349**	0.0547	0.1856
Campaigns for healthy eating	**0.4805**	0.0705	0.4636
Banning unhealthy food (fast food) and soft drinks from schools	0.3131	**0.5223**	0.0703
Restricting advertisements for unhealthy food	0.1062	**0.7973**	0.1015
Restricting advertisements for unhealthy food on children’s TV channels	0.1095	**0.7670**	0.0418
Banning of misleading advertisements	0.0956	**0.7684**	0.0986
Health care insurance bonus for active/health beneficial activites	0.1340	0.0510	**0.6833**
Financial support/subvention of gym classes	0.1001	0.0651	**0.8072**
Government based offers for active lifestyles	0.1562	0.1193	**0.6715**
Eigenvalues	1.64	3.37	1.04
% of accounted variance	17.90	19.89	17.25

Varimax rotated factor loadings of 3 factors with Eigenvalue>1 (n = 971).

**Table 5:** Factor scores regressed on sociodemographic and illness related variables.

Variable	Factor 1 (healthy eating promotion,mean agreement score)	Factor 2 (restriction, meanagreement score)	Factor 3 (financial, governmentalregulation, mean agreement score)
Female	0.15** (0.05–0.26)	0.09** (0.02–0.16)	0.27*** (0.17–0.37)
Age (years)	0.01*** (0.007–0.012)	0.001* (0.000–0.004)	−0.002* (−0.006–(−0.0001))
External causation (mean agreement score)	0.20*** (0.11–0.30)	0.20*** (0.14–0.26)	0.13** (0.04–0.22)
Internal causation (mean agreement score)	0.06 (−0.04–0.15)	0.07* (0.01–0.13)	0.10* (0.01–0.19)
Genetic causation (mean agreement score)	−0.01 (−0.07–0.54)	−0.02 (−0.06–0.12)	0.01 (−0.04–0.07)
Living in Eastern part of Germany	0.19** (0.05–0.32)	0.02 (−0.07–0.11)	0.23*** (0.10–0.36)
BMI (continuous)	0.01 (−0.002–0.020)	−0.002 (−0.010–0.005)	−0.002 (−0.013–0.008)
High school education(12 yrs vs. less)	0.02 (−0.04–0.09)	−0.002 (−0.04–0.04)	−0.04 (−0.10–0.02)
Overweight partner (yes/no)	0.15 (−0.04–0.34)	−0.03 (−0.15–0.09)	0.002 (−0.18–0.18)
Problem solution^a^	−0.01 (−0.019–0.004)	−0.0004 (−0.008–0.007)	0.009 (−0.002–0.199)
Stigmatizing attitudes(FPS, mean score)	0.08 (−0.04–0.20)	0.07 (−0.006–0.143)	0.19*** (0.08–0.30)
Constant	1.99*** (1.34–2.65)	3.20*** (2.78–3.62)	2.46*** (1.85–3.06)
Pseudo R^2^ (%)	10.0	9.0	8.5

All variables simultaneously introduced, full linear regression models; regression coefficients (B) and confidence intervals in parentheses, n = 955; *p<0.05, **p<0.01, ***p<0.001.

a “Obesity is a problem that has to be solved individually ( = 1) or on a societal level ( = 5)”; FPS – Fat Phobia Scale.

## Discussion

Our results showed that the general population of Germany seems to be generally very open-minded towards obesity prevention. This finding, however, indicates that the German population also sees obesity as a modifiable and self-caused condition. Respondents spontaneously named efforts aimed at the individuals most often and almost exclusively. This attribution to individuals has been shown before for Germany [23]; [24] and world-wide [14]. Specific ideas on how to modulate these efforts within individuals (such as how to eat in a more healthy way) are not mentioned by the respondents in our sample; it seems that there is a general idea of eating being a problem in the development of obesity rather than generalizing this idea to specific prevention efforts. Our question obviously was not able to capture the opinion of the general public on how to persuade people to eat healthier.

General support of obesity prevention seems to present a general attitude within the population since it was not associated with socio-demographic or condition-specific variables, however, the slightly higher education in our sample needs to be taken into account. It has been shown that lower education is associated with higher prevalence rates of obesity [25] which might in part relate to a lower level of knowledge about the condition and its aetiology. These factors may be in turn associated with a higher awareness and therefore higher prevention support. We might therefore over-estimate prevention support in regard to the German population.

Only higher stigmatizing attitudes led to lower prevention support. Higher stigmatizing attitudes summarized attributes such as lazy and without will-power. One could hypothesize that a tendency to classify obese individuals as such, leads to less expectations of prevention success and is therefore negated. It has to be pointed out, though, that case numbers for not finding obesity prevention possible are very low (1.76%).

Although the readiness to take part in preventive programs did not differ in overweight individuals compared to their normal-weight counterparts, higher BMI showed a significant association to higher stated readiness. Post-hoc analysis showed that obesity (BMI≥30) was associated with an even higher willingness to partake in preventive programs (p = 0.05); an indicator the greater suffering experienced by obese individuals (compared to those with only overweight). An effect of education was not found. Previous studies on prevention support, however indicate that lower education increases the willingness to participate in prevention programs [26]. This effect might have not been detected since this sample contained a higher number of higher educated respondents. Furthermore, higher age of the respondent led to lower willingness to participate which might reflect a lower perceived necessity. Compared to younger respondents, willingness was lowest in the oldest age group (over 81 years of age). One could assume a tendency to expect failure in these prevention efforts that comes with higher age.

An attribution of obesity to genetic causes was associated with higher willingness to take part in preventive programs. It seems here that the general public is indeed sensitive for the meaning of genetic predispositions and understands that individual or environmental prevention is especially crucial in those individuals.

An interplay of genetic predisposition and environmental as well as behavioral choices is believed to account for parts of the obesity epidemic [27]. Concordantly, an overwhelming amount of participants stated that they would at least partially pay for these programs themselves (86.9%). Higher age was associated with higher willingness to pay. These numbers exceed previous research substantially. In a German study, about 60% were willing to pay some amount (mainly up to 20€) [28]. Even compared investigation of other illnesses in German samples, such as depression [26], willingness to pay was much higher in this sample. This finding might reflect the higher media coverage and information campaigning regarding obesity that has occurred over the last few years. Likewise, the negative depiction of obese individuals in the media [29] might have enhanced the will to stay thin in order to bypass stigmatization and negative health consequences. One group of authors even entitled their article on weight stigma in the media “Norwegians fear fatness more than anything else” [30]. Furthermore, it was shown that greater fear of fat does predict greater weight loss [31].

On another note, the German health care and social security systems have undergone transformation, emphasizing the role of individual prevention and responsibility. It seems possible that this has led to greater willingness for self-paid measures within the population.

In regard to specific prevention strategies, those strategies with a focus on healthy eating receive the highest support rates. Enhanced nutrition labeling, which is already in place in Germany, is supported by the majority of the respondents. Additionally to labeling individual products, consumer oriented websites have been established in Germany, combating delusive advertisement and product naming [32]. Two recent reviews, however, limit expectation on effectiveness of mere nutrition labeling regarding consumer’s dietary pattern [33]; [34]. A recent study, however, finds traffic light visual labeling to be an effective and cost-saving measure in obesity prevention [35], making this strategy a generally supported and effective prevention effort.

Even after factor analysis, the “healthy eating promotion”-factor represents the most popular measures. Access to healthy foods has been discussed as one important factor when trying to change the obesogenic environment. It seems interesting that campaigning on healthy foods is seen potentially effective, as previous studies were able to point out that the association of socio-economic status and obesity is linked to a missing access to certain food and to a lack of information on healthy food [25]; [36]. These obstacles certainly need to be tackled and are obviously approved of by the general public.

External causation (such as bad food environment) is associated to higher support of this kind of measure as it is with all three factors. This finding is not surprising, since we specifically named structural preventive measures. Respondents that think of obesity as environmentally caused are naturally more likely to support the alteration of these factors. Restrictive measures that include laws to prohibit certain types of advertisement and regulative measures find higher approval rates in the former Eastern part of Germany. A stronger, centralized government that acts market-regulating has been experienced by those respondents and might therefore still be supported. Overall prevention support has been linked to residence in eastern Germany before [28] and still seems to differ from the former western part.

Stigmatizing attitudes only showed a significant association with the monetary regulation measures. Higher stigmatizing attitudes resulted in higher support of receiving financial benefits compared to obese individuals (e.g. health care insurance bonus). If we had reversed the items (e.g. higher insurance fees for the obese) we most likely would have found the same result pattern, indicating a general opinion of not wanting to pay for the self-inflicted condition of others. The same discussion has evolved around nicotine and alcohol abuses. These examples show that the belief of self-determination, potentially equivalent to the classic protestant work ethics [37], are center of a wider ethical discussion that health care systems are faced with. Especially with obesity, policy makers are obliged to point out the multi-facetted origins of the condition.

### Limitations

Some limitations of this survey need to be taken into account. First of all, the overall response rate was at 50.9%; this number, however has been shown to be typical in telephone surveys [38]. Furthermore, the sample contained slightly older and better educated persons compared to the general population, and accordingly, a sampling bias cannot be ruled out. This survey could not cover questions on other substantial areas of prevention (such as international agencies, health care sector) and did not cover all proposed core action fields for governments [9]. Future surveys ought to focus on those fields. In light of recent efforts of European countries to introduce taxation of unhealthy foods (as done in Denmark, taxing saturated fatty acids), support for this kind of measure would have been helpful. Taxing unhealthy food (rather than lowering costs for healthy foods) has shown to have a positive impact on consuming behavior in experimental studies before and might be another possible point of market regulation [35]; [39]. Furthermore, the authors are aware that a proclaimed intention to pay for prevention programs does not necessarily result in actual behavior. Lastly, our results are restricted by the fair amount of variance all models were able to explain. Obviously, additional factors that were not examined in the current study are of high influence on the variables investigated.

### Conclusions

Structural prevention efforts are supported by the majority of the general public in Germany. As these are the only measures that have shown to be cost-effective in a recent analysis [40], the German population seems willing to support necessary steps of action. A libertarian paternalism as proposed might be a supported strategy. Surprisingly, the vast majority proclaims willingness to pay themselves for programs of weight gain prevention. For Germany, the government and communities ought to be encouraged by these results to start the implementation of structural obesity prevention.
